# Prevalence, risk factors, and neurobehavioral comorbidities of epilepsy in Kenyan children

**DOI:** 10.1002/epi4.12069

**Published:** 2017-08-19

**Authors:** Charles J. Kind, Charles R. J. C. Newton, Symon M. Kariuki, the Neurodevelopment Disorders study group, Amina Abubakar, Fredrick Ibinda, Joseph Gona, Rachael Odhiambo, Martha Kombe, Michael Kazungu, Jacqueline Philips‐Owen

**Affiliations:** ^1^ St. Johns College University of Oxford Oxford United Kingdom; ^2^ KEMRI‐Wellcome Trust Research Programme Kilifi Kenya; ^3^ Department of Psychiatry University of Oxford Oxford United Kingdom

**Keywords:** Convulsive epilepsy, Prevalence, Neurobehavioral comorbidities, Children, Africa

## Abstract

**Objective:**

To investigate the prevalence, risk factors, clinical features, and neurobehavioral comorbidities of epilepsy and acute symptomatic seizures in school‐aged children in Kilifi, Kenya.

**Methods:**

Randomly selected children (N = 11,223) were screened for epilepsy and other neurodevelopmental disorders. Those who screened positive were invited for further clinical, electroencephalographic (EEG), and neuropsychological evaluations. Prevalence was measured by dividing cases by screened population, providing Agresti–Coull confidence intervals (CIs). Prevalence ratios were computed using log binomial regression, and odds ratios (ORs) were computed using logistic regression; both were implemented with generalized linear models. Attention‐deficit hyperactivity disorder (ADHD), autism spectrum disorder (ASD), and other neurodevelopmental impairments were assessed in cases and controls.

**Results:**

Prevalence of lifetime epilepsy was 20.9 per 1,000 (95% CI = 18.4–23.7), and that of active epilepsy was 11.5 per 1,000 (95% CI = 9.7–13.6). Prevalence of acute symptomatic seizures was 68.8 per 1,000 (95% CI = 64.2–73.6). Acute symptomatic seizures preceded a diagnosis of epilepsy in 8% of children. Of 98 children diagnosed with epilepsy, focal seizures were seen in 79%, abnormal EEG was seen in 39%, and 83% were not receiving antiepileptic drugs. Childhood absence epilepsy and Lennox–Gastaut epilepsy were the most easily identifiable epilepsy syndromes. Perinatal complications, previous hospitalization, geophagia, and snoring were risk factors for epilepsy. Family history of seizures, abnormal pregnancy, previous hospitalization, and snoring were risk factors for acute symptomatic seizures. Neurobehavioral comorbidities were present in 54% of subjects with lifetime epilepsy and in 3% of controls, with associations for individual comorbidities being statistically significant: ADHD (OR = 14.55, 95% CI = 7.54–28.06), ASD (OR = 36.83, 95% CI = 7.97–170.14), and cognitive impairments (OR = 14.55, 95% CI = 3.52–60.14).

**Significance:**

The burden of seizure disorders in this area is higher than in locations in high‐income countries, and can be reduced by preventing risk factors. A comprehensive management plan for neurobehavioral comorbidities of epilepsy should be incorporated into standard epilepsy care.

**Key Points**

- Epilepsy occurred in 21 of 1,000 Kenyan children (aged 6–9 years), and acute symptomatic seizures occurred in 69 of 1,000
- Acute symptomatic seizures preceded a diagnosis of epilepsy in 8% of children, and the two conditions had shared risk factors, particularly previous hospitalization with nontraumatic acute encephalopathy
- Focal features occur in about 79% of Kenyan children with epilepsy; childhood absence and Lennox–Gastaut epilepsy are the most easily identifiable epilepsy syndromes
- Epilepsy in older children is associated with neurobehavioral comorbidities such as attention‐deficit hyperactivity disorder and autism spectrum disorders

Epilepsy is common in sub‐Saharan Africa,^1^ where it exerts an enormous burden on the health system,^2^ and is associated with substantial mortality relative to elsewhere in the world.^3^ Children bear the largest burden of the disease.^4^ Despite this, only a small number of epidemiological studies of epilepsy in children have been carried out, with data for the most recent study collected more than a decade ago.^5^ Additionally, no study has attempted to describe epilepsy syndromes, and there are few studies of the comorbidity with other neurodisabilities in African children, despite evidence for their existence.^6^, ^7^

Studies reporting on the burden of epilepsy in African children range between 7.3 per 1,000 in South Africa^8^ and 41 per 1,000 in Kenya.^5^ Although wide variation in estimates may be attributed to differences in risk factors within the sites, it may also be due to methodological differences. Additional risk factors for childhood mental health problems (e.g., snoring and geophagia) have been documented in recent studies,^9^ but it is unclear whether they are important in children with epilepsy. We conducted an epidemiological study that employed sensitive screening methods to estimate the prevalence of epilepsy and its associated risk factors in children from a rural area in Kenya. Clinical and electroencephalographic features of epilepsy were characterized, and neurobehavioral comorbidities were described by trained clinicians. We recognized that acute symptomatic seizures should be systematically screened, as they can easily be misdiagnosed as epilepsy, overestimating the burden. These data are required to inform decisions on comprehensive preventative and therapeutic interventions, aimed at reducing the large treatment gap for epilepsy in children.

## Methods

### Study site and population

This study was conducted on the Kenyan coast in a defined area known as the Kilifi Health and Demographic Surveillance System (KHDSS), which is located in Kilifi County.^10^ KHDSS is hosted at KEMRI‐Wellcome Trust Research Programme, which is located about 60 km north of Mombasa. The main population in KHDSS is the Mijikenda ethnic group, the majority of whom are subsistence farmers. The literacy levels are low, and Kilifi County is one of the poorest administrative regions in Kenya. There is one epilepsy and neurodevelopmental clinic run by researchers at KEMRI‐Wellcome Trust Research Programme and Kilifi County Hospital (KCH).

### Sampling

This study involved children aged 6–9 years living within KHDSS, who form a total population of about 28,000. The age group of 6–9 years was chosen because febrile seizures are uncommon in this group, so they would not confound epilepsy estimates, and to allow for comparison with a previous study conducted around 10 years ago that used the same age group.^5^ We had calculated that screening about 15,000 randomly selected subjects from the 28,000 children will detect epilepsy and acute symptomatic seizures with a precision or margin of error of <1%, assuming a prevalence of seizure disorders of 5% in the community. The RAND() command of MySQL was used to select the eligible children using a simple random sampling method.

### Study design and procedures

This was a two‐stage design. Stage I involved screening of epilepsy and other neurodevelopmental disorders in the community. Those who screened positive in stage I were invited for further clinical assessment in stage II. Neurodevelopmental Screening Tool (NDST) was used for screening in stage I and was administered to the parents or close caregivers of the eligible child.^11^ The questionnaire has one seizure question (Since birth, does the child sometimes have fits, become rigid, or lose consciousness?), but those screening positive for other neurodevelopmental questions were also assessed for epilepsy and acute symptomatic seizures in stage II. According to a pilot study nested in this main epidemiological survey, the NDST detected epilepsy with a sensitivity of 95% (95% confidence interval [CI] = 94–96%). The negative predictive value (the probability that those screening negative did not truly have a seizure disorder) for the NDST was excellent (99.0%, 95% CI = 98.6–99.5%), although in stage II the clinician still saw a proportion of those who screened negative in stage I to confirm the seizure status.

In stage II, trained clinicians and neuropsychological assessors took a clinical history that included presence of seizures, duration, onset, frequency, and types and use of antiepileptic drugs (AEDs). Cognition was measured with Ravens Matrix Test, attention‐deficit hyperactivity disorder (ADHD) with Kiddie Schedule for Affective Disorders and Schizophrenia, and autism with Autism Diagnostic Observation Schedule. All the tools including NDST were translated into the local language, Kiswahili, through a standardized forward and back translation process. Focused group discussions and in‐depth interviews were conducted with adults in the community to elicit phrases and idioms to be used in the translated version. They were piloted to test their appropriateness in assessing neurodevelopment and adapted accordingly before use in the epidemiological survey. The tools were administered by experienced and trained neuropsychological assessors supervised by a psychologist.

A risk factor questionnaire was given to the parents of every child assessed at stage II, including randomly selected age‐matched controls. The risk factor questionnaire had items on socioeconomic status, medical history, and child habits. The questionnaire was designed for use in neurodevelopmental studies following a thorough review for possible risk factors for neurodevelopment. Electroencephalography was performed according to the international 10–20 system, as previously described.^12^

We classified seizures as focal, generalized, or others as recommended by the position paper of the International League against Epilepsy (ILAE) commission for Classification and Terminology recently published in *Epilepsia*.^13^ Parents were asked to describe the three most recent seizure types, so that each child could be categorized for more than one seizure type, if appropriate. Seizure frequency was categorized into daily, weekly, monthly, and yearly. Status epilepticus was defined as seizures lasting for 30 min or intermittent seizures for a period of 30 min as timed by a watch or, for those without watches, events of that approximate duration such as boiling a pot of maize, a radio news bulletin, or milking a cow.^14^

### Diagnosis and definitions

Epilepsy was defined as a history of two or more unprovoked seizures according to recommendations by the ILAE.^15^ Those with unprovoked seizures in the past 12 months were deemed to have active epilepsy, whereas lifetime epilepsy included a history of unprovoked seizures occurring during the entire life of the child, up to 9 years. Acute symptomatic seizures were defined as seizures provoked by a febrile illness, as previously described.^16^, ^17^ Cognition was considered impaired if the child had a standardized z score of <2. Motor deficits were present if a child was unable to hold implements, and unable to sit, stand upright, and put on clothes if of appropriate age. Prevalence was operationally defined as the proportion of children aged 6–9 years with a history of epilepsy as of the day the epidemiological survey was conducted.

### Statistical analysis

Data were entered using MySQL, and analyzed using STATA software (version 13.1, Stata Corp, College Station, TX, U.S.A.). Prevalence was computed by dividing observed cases by the total population; CIs were based on the Agresti–Coull interval approach, which has a coverage probability that is close to nominal confidence level, and does not produce narrow intervals as the exact or Wald intervals approach would with large sample sizes such as in this study.^18^ The prevalence accounted for sensitivity of the screening tools at stage I, and for attrition (proportion of those who fail to turn up in stage II for further clinical evaluation, following up to three invitations) between stage I and II, by dividing crude prevalence with sensitivity and attrition (both expressed as proportions). Log binomial regression was used to compute prevalence ratios for prevalence estimates by sex and age group. Generalized linear models with a logit were used to compute the odds ratios (ORs) of the factors associated with either epilepsy or acute symptomatic seizures. Discrete variables were compared using Pearson chi‐square, or Fisher exact test where observations in a cell were sparse (<5). Bonferroni correction was used to adjust for multiple testing in risk factor analysis.

### Ethical approval

Permission to conduct this study was obtained from the Scientific and Ethics Review unit of KEMRI. Parents of children gave informed consent for their children to participate.

## Results

A total of 11,223 children aged 6–9 years were initially screened, of whom 2,361 (21.0%) screened positive according to the NDST in stage I screening. Of these, 1,640 (69.5%) were seen by a clinician who assessed them as having epilepsy, acute symptomatic seizures, or neither. Those who did not attend the assessment were similar to those who did in terms of age (p = 0.367) and sex (p = 0.087). Sociodemographic comparisons between the attendance groups cannot be made because this information was only collected at the clinical assessment stage.

Of the 1,640 children seen by the clinician in stage II, 556 (33.9%) were positive for the specific seizure question in stage I. The total number of children diagnosed with epilepsy was 98 (Fig. S1); of these, active epilepsy was seen in 54 (55%). Of the 98 cases of epilepsy, 69 (70.4%) were generated from those who screened positive in the NDST specific seizure question, whereas the remainder were from clinical evaluation of those who screened positive for other NDST developmental disorders. After accounting for attrition between stages I and II, and sensitivity of the screening tools, the adjusted total number of children with epilepsy was 234, 129 of whom had active epilepsy. The distribution of epilepsy cases in those who screened positive and in those who screened negative is shown in Fig. S1. The adjusted prevalence of lifetime epilepsy was 20.9 per 1,000 (95% CI = 18.4–23.7), and that of active epilepsy was 11.5 per 1,000 (95% CI = 9.7–13.6). The distribution of prevalence by age and sex is shown in Table 1. There were no significant differences in prevalence of epilepsy between age groups or sex, according to the computed prevalence ratios.

**Table 1:** Prevalence of epilepsy and acute symptomatic seizures in the year 2015, by sex and age

Age, yr	Number screened	Number with lifetime epilepsy	Number with active epilepsy	Number with acute symptomatic seizures	Prevalence lifetime epilepsy per 1,000 (CI)	Prevalence active epilepsy per 1,000 (CI)	Prevalence ratio for epilepsy^a^^,^ b	Prevalence acute symptomatic seizures per 1,000 (CI)	Prevalence ratio for acute symptomatic seizures
6	1,651	10	10	42	6.1 (3.1–11.2)	6.1 (3.1–11.2)	1.00	25.4 (18.7–34.2)	1.00
7	3,803	29	19	98	7.6 (5.2–10.9)	5.0 (3.1–7.8)	1.26 (0.62–2.58)	25.7 (21.1–31.3)	1.01 (0.71–1.45)
8	3,636	36	14	99	9.9 (7.1–13.7)	3.9 (2.2–6.5)	1.64 (0.81–3.29)	27.2 (22.3–33.1)	1.07 (0.75–1.53)
9	2,133	23	11	69	10.8 (7.1–16.2)	5.2 (2.8–9.3)	1.78 (0.85–3.74)	32.3 (25.6–40.8)	1.27 (0.87–1.86)
Sex									
Male	5,646	51	29	180	9.0 (6.9–11.9)	5.1 (3.5–7.4)	1.00	31.9 (27.5–36.8)	1.00
Female	5,577	47	25	128	8.4 (6.3–11.2)	4.5 (3.0–6.6)	1.07 (0.72–1.59)	23.0 (19.3–27.2)	1.39 (1.11–1.74)
Crude total	11,223	98	54	308	8.7 (7.2–10.6)	4.8 (3.7–6.3)	—	27.4 (24.6–30.6)	—
Extrapolated total^c^	11,223	234	129	772	20.9 (18.4–23.7)	11.5 (9.7–13.6)	—	68.8 (64.2–73.6)	—

CI, confidence interval, which was based on the Agresti–Coull interval approach.

a Includes lifetime epilepsy only.

b Prevalence ratio computed with log binomial regression. Baseline categories for age and sex are 6 years and male sex, respectively.

c Adjusted for attrition and screening sensitivity.

The total number of children with acute symptomatic seizures was 308 (Fig. S1). The adjusted prevalence was 68.8 per 1,000 (95% CI = 64.2–73.6; Table 1). There were no significant differences in prevalence of acute symptomatic seizures between age groups, but prevalence was significantly greater in males than females. Acute symptomatic seizure preceded the diagnosis of epilepsy in 23 of 98 (7.5%) children with epilepsy. Of the 308 cases of acute symptomatic seizures, 204 (66.2%) were treated at KCH in the last 6–9 years, with 124 (40.3%) treated for acute symptomatic seizures. Based on the hospital records for these 124 cases treated for acute symptomatic seizures, the most important diagnoses associated with acute symptomatic seizures were falciparum malaria in 62 (50%), respiratory tract infections in 38 (30.7%), seizures with fever in 28 (22.5%), and gastroenteritis in 16 (12.9%). About one‐quarter (24.2%) of acute symptomatic seizures treated at the hospital were repetitive, whereas 16.1% were focal.

### Clinical features of epilepsy

Of 98 children diagnosed with epilepsy in stage II, focal (78.6%) and generalized seizures (70.4%) described any of the three most recent episodes of seizures (not mutually exclusive). Generalized seizures were more common in active (81.5%) than inactive epilepsy (56.8%; p = 0.008), and generalized tonic–clonic seizures were the most common (57.1%; Table 2). Of all the focal seizure types, the most common was focal impaired awareness (76.5%); there were significantly more of these in active (90.7%) than inactive epilepsy (59.7%; p < 0.0001). Other significant differences in seizure types between active and inactive epilepsy are shown in Table 2.

**Table 2:** Clinical features of seizures in those diagnosed with epilepsy

Dominant seizure types	Lifetime epilepsy, n (%); N = 98	Active epilepsy, n (%); N = 54	Inactive epilepsy, n (%); N = 44	χ^2^ p^a^
Generalized seizure types				
Any generalized seizure^b^	69 (70.4)	44 (81.5)	25 (56.8)	0.008
Generalized tonic–clonic	56 (57.1)	34 (63.0)	22 (50.0)	0.197
Generalized other motor^c^	45 (45.9)	25 (46.3)	20 (45.5)	0.934
Generalized nonmotor/absence	20 (20.4)	17 (1.5)	3 (6.8)	0.003^d^
Focal seizure types				
Any focal seizure	77 (78.6)	50 (92.6)	27 (61.6)	<0.001^d^
Focal motor	46 (46.9)	27 (50.0)	19 (43.2)	0.501
Focal impaired awareness	75 (76.5)	49 (90.7)	26 (59.1)	<0.001
Focal sensory	15 (15.3)	12 (22.2)	3 (6.8)	0.048^d^
Focal to bilateral tonic–clonic	5 (5.1)	2 (3.7)	3 (6.8)	0.654^d^
Impaired awareness	27 (27.6)	24 (44.4)	3 (6.8)	<0.001^d^
Other clinical features				
Seizure frequency^e^				
Daily	17 (17.4)	14 (25.9)	3 (6.8)	0.016^d^
Weekly	30 (30.6)	23 (42.6)	7 (15.9)	0.004
Monthly	57 (57.5)	35 (63.6)	22 (50.0)	0.173
Yearly	42 (42.9)	20 (37.0)	22 (50.0)	0.197
Status epilepticus	26 (26.5)	17 (31.5)	9 (20.5)	0.219
Self‐reported AED use	13 (17.1)	8 (19.1)	5 (14.7)	0.617

AED, antiepileptic drug.

a Comparisons are between active and inactive epilepsy.

b Not including focal to bilateral tonic–clonic seizures.

c Generalized other motor seizures comprise clonic, tonic, myoclonic, atonic, myoclonic–atonic, and epileptic spasms; focal motor seizures comprise automatisms and clonic, myoclonic, tonic, atonic, hyperkinetic, and epileptic spasms; generalized nonmotor/absence seizures comprise typical, atypical, myoclonic, and eyelid myoclonia. All percentages are derived from observed numbers divided by the totals in the column heading. Seizure types are not mutually exclusive, because a child can have different seizure types across multiple episodes of seizures.

d Fisher exact test used when any value for this variable was <5.

e Seizure frequency categories are not mutually exclusive, apart from yearly, which includes all those that are not included in the other three categories.

Of those with epilepsy, self‐reported AED use was reported in 13 (17.1%), daily seizures in 17 (17.4%), and status epilepticus in 26 (26.5%; Table 2). As would be expected, daily (25.9% vs. 6.8%, p = 0.016) and weekly seizures (42.3% vs. 15.9%, p = 0.004) were significantly more frequent in active than inactive epilepsy. Self‐reported nontreatment was not significantly more frequent in status epilepticus (p = 0.295) or daily seizures (p = 0.324) than those without these features. The age at onset of seizures among those with epilepsy was 3 years (interquartile range = 1–6), and it was similar between active epilepsy and inactive epilepsy. Age at onset of seizures was inversely correlated with self‐reported use of AED (ρ = −0.28, p = 0.013), but not with status epilepticus (p = 0.465), daily seizures (p = 0.684), or weekly seizures (p = 0.520).

### Electroencephalographic features

Sixty‐four out of the 98 (65.3%) children with epilepsy attended clinic for an electroencephalographic (EEG) recording. There were no differences between those who attended and those who did not attend the EEG recording in terms of age (p = 0.918), sex (p = 0.990), status epilepticus or self‐reported AED use, parental marital status (p = 0.107), maternal schooling (p = 0.138), maternal economic activity (p = 0.731), paternal schooling (p = 0.324), and paternal economic activity (p = 0.797). People with active epilepsy were more likely to attend the clinic for the EEG than those with inactive epilepsy (76% vs. 50%, p = 0.006; Table 2).

Abnormal EEGs were recorded in more than one‐third of both lifetime and active epilepsy cases (Table S1). Generalized epileptiform discharges were the most common abnormality. There were no significant differences between cases of active and inactive epilepsy in terms of EEG abnormalities. Focal epileptiform discharges were observed in 13% of those with a generalized seizure semiology, but the occurrence was not different from those without these seizures types (11%; p = 0.680).

Some EEG traces displayed patterns characteristic of well‐described epilepsy syndromes (Fig. S2). Three children (0.05%) had childhood absence syndrome, two (0.03%) had benign epilepsy of childhood with centrotemporal spikes, and one (0.02%) had temporal lobe epilepsy.

### Risk factors for epilepsy and acute symptomatic seizures

The risk factors for lifetime and active epilepsy and acute symptomatic seizures were compared to 471 controls who screened negative for seizures in stage I. Multivariate logistic regression analysis (Table 3) identified perinatal complications, previous hospital admissions, eating soil, and snoring as risk factors for epilepsy. The same model (Table 4) identified a family history of seizures, abnormal pregnancy, previous hospital admissions, and snoring as risk factors for acute symptomatic seizures. The father having attended school was significantly protective against acute symptomatic seizures.

**Table 3:** Uni‐ and multivariate ORs of risk factors for epilepsy

Risk factor	Controls, n = 471	Epilepsy, n = 98^a^	Univariate OR(95% CI)^b^	Multivariate OR (95% CI)^c^
Median (range) age of child, yr	8 (6–9)	8 (6–9)	1.21 (0.95–1.53)*	1.28 (0.97–1.68)
Male sex				
No	243 (51.6)	47 (47.9)	1.00	
Yes	228 (48.4)	51 (52.0)	1.16 (0.75–1.79)	1.18 (0.69–2.03)
Median (range) age of mother at birth, yr	27 (15–46)	26 (13–41)	0.98 (0.95–1.01)	—
Mother's marital status^d^				
Never married/single	13 (2.8)	9 (9.3)	1.00	
Divorced/separated	48 (10.3)	12 (12.37)	0.36 (0.13–1.04)	
Widowed	27 (5.8)	10 (10.31)	0.53 (0.17–1.64)	
Married	376 (81.0)	66 (68.0)	**0.25 (0.10**–**0.62)**	0.83 (0.59–1.16)
Mother attended school				
No	165 (35.8)	38 (38.8)	1.00	
Yes	296 (64.2)	60 (61.2)	0.88 (0.56–1.38)	—
Father attended school				
No	29 (6.8)	10 (10.9)	1.00	
Yes	398 (93.2)	82 (89.1)	0.60 (0.28–1.27)	—
Mother earns cash				
No	88 (19.1)	15 (15.5)	1.00	
Yes	373 (80.9)	82 (84.5)	1.28 (0.71–2.34)	—
Father earns cash				
No	29 (6.6)	12 (13.3)	1.00	
Yes	414 (93.5)	78 (86.7)	**0.46 (0.22**–**0.93)**	0.64 (0.25–1.59)
Median (range) number of children ever born	6 (1–12)	6 (1–11)	0.95 (0.87–1.03)*	—
Father dead				
No	431 (93.3)	88 (89.8)	1.58 (0.75–3.34)*	
Yes	31 (6.7)	10 (10.2)		—
House status				
Completely dilapidated shack	28 (6)	1 (1.0)	1.00	
Needs major repairs	67 (14.4)	23 (23.5)	**9.6 (1.24**–**74.81)**	
Incompletely constructed	75 (16.1)	13 (13.3)	4.85 (0.61–38.91)	
Needs no or minor repairs	297 (63.6)	61 (62.2)	**5.75 (0.77**–**43.15)**	0.96 (0.73–1.25)
Family history of seizures				
No	395 (84.6)	65 (67.0)	1.00	
Yes	72 (15.4)	32 (33.0)	**2.70 (1.65**–**4.42)**	1.76 (0.97–3.19)
Pregnancy				
Normal	418 (90.9)	79 (81.4)	1.00	
Abnormal	42 (9.1)	18 (18.6)	**2.27 (1.24**–**4.14)**	1.24 (0.58–2.67)
Place of delivery				
Hospital/clinic	119 (25.9)	28 (28.9)	1.00	
Home	339 (74.0)	69 (71.1)	0.87 (0.53–1.41)	—
Perinatal complications^e^				
No	433 (96.0)	82 (85.4)	1.00	
Yes	18 (4.0)	14 (14.6)	**4.11 (1.96**–**8.59)**	***2.88 (1.20***–***6.92)***
Median (range) duration of breast‐feeding, mo	24 (6–36)	24 (2–38)	1.01 (0.98–1.04)	—
Child has previous hospital admission(s)				
No	395 (86.1)	53 (54.1)	1.00	
Yes	64 (14.0)	45 (45.9)	**5.24 (3.25**–**8.45)**	***5.81 (3.28***–***10.28)***
Child has previous head injury				
No	440 (96.3)	90 (92.8)		
Yes	17 (3.7)	7 (7.2)	2.01 (0.81–4.99)*	—
Child eats cassava				
No	9 (1.9)	4 (4.1)	1.00	
Yes	459 (98.1)	94 (95.9)	0.46 (0.14–1.53)*	—
Pets in the household^f^				
No	253 (54.1)	45 (46.4)	1.00	
Yes	215 (45.9)	52 (53.6)	1.36 (0.88–2.11)*	—
Child eats soil				
No	448 (96.6)	84 (86.6)	1.00	
Yes	16 (3.5)	13 (13.4)	**4.33 (2.01**–**9.35)**	***4.27 (1.61***–***11.32)***
Child snores >3 nights per week				
No	316 (68.3)	36 (37.1)	1.00	
Yes	147 (31.8)	61 (62.9)	**3.64 (2.31**–**5.75)**	***4.16 (2.37***–***7.31)***
Childs uses bed net				
No	36 (7.7)	4 (4.1)		
Yes	431 (92.3)	94 (95.9)	1.96 (0.68–5.65)*	—
Median (range) birth weight, kg	3.4 (2.0–4.7)	3.4 (1.0–2.9)	1.00 (0.99–1.01)	—

Values are n (%) unless otherwise indicated.

CI, confidence interval; OR, odds ratio.

a Includes lifetime and active epilepsy.

b p ≤ 0.25 is indicated by asterisk, p ≤ 0.10 is indicated by bold, and added to the multivariate model. OR was computed using logistic regression (generalized linear model with logit link).

c Age and sex were added a priori to the multivariate model irrespective of the significance level. Significant multivariate associations are indicated by bold italics. For variables with multiple categories, the OR represents all categories combined.

d The three categories of mother's marital status are compared to the baseline category of never married/single.

e Includes difficulties in child's breathing, crying, and breast‐feeding.

f Includes dogs and cats.

**Table 4:** Uni‐ and multivariate ORs of risk factors for acute symptomatic seizures

Risk factor	Controls, n = 471	Acute symptomatic seizures, n = 308	Univariate OR(95% CI)^a^	Multivariate OR(95% CI)^b^
Median (range) age of child, yr	8 (6–9)	8 (6–9)	1.07 (0.92–1.25)	1.17 (0.96–1.41)
Male sex				
No	243 (51.6)	128 (41.6)	1.00	
Yes	228 (48.4)	180 (58.4)	**1.50 (1.12**–**2.00)**	1.43 (0.99–2.04)
Median (range) age of mother at birth, yr	27 (15–46)	26 (15–46)	0.99 (0.97–1.01)	—
Mother's marital status^c^				
Never married/single	13 (2.8)	10 (3.3)	1.00	
Divorced/separated	48 (10.3)	31 (10.1)	0.84 (0.33–2.15)	
Widowed	27 (5.8)	23 (7.5)	1.11 (0.41–2.99)	
Married	376 (81.0)	242 (79.1)	0.84 (0.36–1.94)	—
Mother attended school				
No	165 (35.8)	106 (34.6)	1.00	
Yes	296 (64.2)	200 (65.4)	1.05 (0.78–1.42)	—
Father attended school				
No	29 (6.8)	33 (11.1)	1.00	
Yes	398 (93.2)	265 (88.9)	**0.59 (0.35**–**0.99)**	***0.41 (0.22***–***0.75)***
Mother employed				
No	88 (19.1)	46 (15.1)	1.00	
Yes	373 (80.9)	259 (84.9)	1.33 (0.90–1.96)*	—
Father employed				
No	29 (6.6)	27 (9.2)	1.00	
Yes	414 (93.5)	268 (90.9)	0.70 (0.40–1.20)*	—
Median (range) number of children ever born	6 (1–12)	6 (1–13)	0.99 (0.94–1.05)	—
Father dead				
No	431 (93.3)	278 (91.2)	1.00	
Yes	31 (6.7)	27 (8.9)	1.35 (0.79–2.31)	—
House status				
Needs no or minor repairs	28 (6)	7 (2.3)	1.00	
Incompletely constructed	67 (14.4)	54 (17.6)	**3.23 (1.31**–**7.95)**	
Needs major repairs	75 (16.1)	64 (20.9)	**3.41 (1.40**–**8.34)**	
Completely dilapidated shack	297 (63.6)	182 (59.3)	**2.45 (1.05**–**5.72)**	1.12 (0.92–1.35)
Family history of seizures				
No	395 (84.6)	213 (70.1)	1.00	
Yes	72 (15.4)	91 (29.9)	**2.34 (1.65**–**3.33)**	***2.05 (1.36***–***3.09)***
Pregnancy				
Normal	418 (90.9)	241 (79.0)	1.00	
Abnormal	42 (9.1)	64(21.0)	**2.64 (1.74**–**4.02)**	***2.09 (1.23***–***3.55)***
Place of delivery				
Hospital/clinic	119 (25.9)	65 (21.2)	1.00	
Home	339 (74.0)	241 (78.8)	1.30 (0.92–1.84)*	—
Perinatal complications^d^				
No	433 (96.0)	274 (91.0)	1.00	
Yes	18 (4.0)	27 (9.0)	**2.37 (1.28**–**4.39)**	***1.73 (0.78**–**3.84**)*
Median (range) duration of breast‐feeding, mo	24 (6–36)	24 (6–41)	1.01 (0.99–1.04)	—
Child has previous hospital admission(s)				
No	395 (86.1)	136 (44.3)	1.00	
Yes	64 (14.0)	171 (55.7)	**7.76 (5.48**–**10.98)**	***7.93 (5.33***–***11.80)***
Child has previous head injury				
No	440 (96.3)	287 (93.8)	1.00	
Yes	17 (3.7)	19 (6.2)	1.71 (0.88–3.35)*	—
Child eats cassava				
No	9 (1.9)	3 (1.0)	1.00	
Yes	459 (98.1)	303 (99.0)	1.98 (0.53–7.38)	—
Pets in the household^e^				
No	253 (54.1)	179 (58.5)	1.00	
Yes	215 (45.9)	127 (41.5)	0.83 (0.62–1.12)*	—
Child eats soil				
No	448 (96.6)	291 (95.1)	1.00	
Yes	16 (3.5)	15 (4.9)	1.44 (0.70–2.96)	—
Child snores >3 nights per week				
No	316 (68.3)	141 (46.2)	1.00	
Yes	147 (31.8)	164 (53.8)	**2.50 (1.86**–**3.37)**	***3.08 (2.13***–***4.47)***
Childs uses bed net				
No	36 (7.7)	15 (4.9)	1.00	
Yes	431 (92.3)	292 (95.1)	1.63 (0.87–3.02)*	—
Median (range) birth weight, kg	3.4 (2.0–4.7)	3.5 (1.5–5.5)	1.00 (0.99–1.00)	—

a p ≤ 0.25 is indicated by asterisk, p ≤ 0.10 is indicated by bold, and added to the multivariate model. OR was computed using logistic regression (generalized linear model with logit link).

b Age and sex were added a priori to the multivariate model irrespective of the significance level. p ≤ 0.05 is indicated by bold italics. For variables with multiple categories, the OR represents all categories combined.

c The three categories of mother's marital status are compared to the baseline category of never married/single.

d Includes difficulties in child's breathing, crying, and breast‐feeding.

e Includes dogs and cats.

### Comorbidities associated with epilepsy

Neurobehavioral and neurological comorbidities were more frequent in those with epilepsy (53 of 98, 54%) than in community controls without neurodisability (22 of 643, 3%, p < 0.0001). Among those with lifetime epilepsy, the association was particularly great for autism spectrum disorder (ASD; OR = 36.83, 95% CI = 7.97–170.14), ADHD (OR = 14.55, 95% CI = 7.54–28.06), cognition (OR = 14.55, 95% CI = 3.52–60.14), and motor impairments (OR = 34.51, 95% CI = 3.77–1,634.62). The ORs were greater in active epilepsy than in lifetime epilepsy, for all comorbidities except cognitive impairments (Table 5). Of the 98 children with epilepsy, 12 (12.2%) had neurobehavioral comorbidities and generalized EEG epileptiform discharges compatible with Lennox–Gastaut syndrome.

**Table 5:** Neurobehavioral comorbidities associated with epilepsy

Comorbidity	Lifetime epilepsy, n (%); N = 98	Active epilepsy, n (%); N = 54	Controls, n (%); N = 643	Adjusted OR, lifetime epilepsy (95% CI)^a^	Adjusted OR, active epilepsy (95% CI)^b^
ADHD	28 (28.6)	18 (33.3)	17 (2.7)	14.55 (7.54–28.06)	17.66 (8.39–37.22)
ASD	11 (11.2)	7 (13.0)	2 (0.3)	36.83 (7.97–170.14)	50.57 (10.01–255.49)
Cognitive impairments	6 (6.1)	2 (3.7)	2 (0.3)	14.55 (3.52–60.14)	6.82 (1.07–43.38)
Motor impairments	5 (5.1)	3 (5.6)	0 (0)	34.51 (3.77–1634.62)^c^	37.76 (2.93–1985.01)^c^
Hearing impairments	1 (1.0)	1 (1.9)	0 (0)	6.90 (0.09–520.29)^c^	11.89 (0.15–934.54)^c^
Visual impairments	2 (2.0)	2 (3.7)	0 (0)	13.38 (0.69–790.16)^c^	24.69 (1.25–1458.66)^c^

ADHD, attention‐deficit hyperactivity disorder; ASD, autism spectrum disorder; CI, confidence interval; OR, odds ratio.

a Association of comorbidities with lifetime epilepsy adjusted for age and sex in a logistic regression model.

b Association of comorbidities with active epilepsy adjusted for age and sex in a logistic regression model.

c Unadjusted OR computed using the immediate command *cci* in Stata, assuming at least one person with comorbidity in controls.

## Discussion

This epidemiological study provides important data on the prevalence of epilepsy and acute symptomatic seizures in school‐aged children, placing them at 21 in 1,000 and 69 in 1,000 of the population, respectively. In those with epilepsy, focal seizures were very common (79%), EEG was abnormal in 39%, and 83% were not receiving an AED. The important risk factors for epilepsy were perinatal complications, previous hospitalization, geophagia, and snoring. More than two‐thirds of acute symptomatic seizures were admitted to a hospital, and falciparum malaria caused half of these seizures. Family history of seizures, previous hospitalization, and snoring were independently associated with acute symptomatic seizures. A number of neurobehavioral abnormalities were strongly associated with epilepsy, notably, ADHD, ASD, and cognitive impairments. Childhood absence epilepsy and Lennox–Gastaut epilepsy were the commonest epilepsy syndromes, as would be expected for this age group.

The prevalence of epilepsy in these children (21 in 1,000) is similar to a study from the U.S.A.,^19^ but higher than the worldwide prevalence estimated in a recent meta‐analysis.^20^, ^21^ Notably, the present study estimates are half what was reported in a previous study in the same area (41 in 1,000), for which data were collected >10 years ago.^5^ Our study included identification of acute symptomatic seizures, which may have improved identification of epilepsy. The widely recognized decline in malaria^22^ since the previous study^5^ may have reduced the burden of epilepsy, which is associated with severe falciparum malaria.^23^ We demonstrated that decline in malaria reduces the incidence of acute symptomatic seizures,^24^ which are associated with epilepsy.^25^ Improved obstetric services and neonatal care, as demonstrated by increased admissions of neonates^26^ and reduced home deliveries compared to the past decade (76% vs. 71%),^5^ may have reduced perinatal complications, further influencing the current prevalence estimate. Conversely, the burden of neurodisabilities (including epilepsy) would be expected to increase with improved childhood survival,^27^ but massive gains in reduction of infectious etiologies of epilepsy such as falciparum malaria^28^ may offset the burden of epilepsy. The prevalence may fail to capture epilepsy in the children who died before the survey (whose seizures likely began in infancy), perhaps explaining the late median age at onset of epilepsy of 3 years.

The prevalence of acute symptomatic seizures in these school‐aged children (6.9%) is higher than those in high‐income countries (2–5%)^29^ and in a population‐based study in Tanzania (2.1%).^30^ The Tanzanian study was conducted in a similar malaria‐endemic region such as ours, and methodological differences may partially explain the variation in estimates; for example, they conducted theirs in an administrative area, whereas we did ours in a demographic area under active surveillance. Additionally, these two rural settings may have different levels of social desirability bias (which can result in underestimation), and epilepsy educational interventions in Kilifi^31^ may have improved the confidence of the community members to report seizures. Acute symptomatic seizures are associated with the risk of epilepsy in this area,^25^ and should be addressed, given that one‐third of these seizures were not treated in a hospital. Control of malaria may reduce the burden of acute symptomatic seizures attributable to malaria.^17^, ^24^

Perinatal and neonatal complications are established risk factors for epilepsy in several studies from Africa,^1^ and may be associated with seizures in the first year of life, which corresponded to the 25th percentile of age at onset of epilepsy. Previous hospitalization was associated with epilepsy probably because it is a proxy for acute infections that are known to cause epilepsy such as cerebral malaria and human immunodeficiency virus (HIV). The association between snoring and epilepsy was reported previously in South Africa,^32^ and is probably related to upper airway obstruction causing hypoxia, which may lower the threshold for seizures. A multivariate reanalysis of snoring as an outcome variable, and acute symptomatic seizures and epilepsy as risk factors, showed no statistically significant associations. This rules out a bidirectional relationship (Table S2), but more studies are needed. An association of epilepsy with eating soil is interesting, and suggests that soil in this area may be contaminated with heavy metals, in particular lead and mercury, which may damage the brain, and should be investigated. Geophagia is also strongly associated with *Toxocara* infection, which was associated with an increased risk of epilepsy in Africa.^1^ The association between geophagia and neurocysticercosis was not supported by previous computed tomography scan and serology studies in this area.^33^, ^34^ However, intellectual disability represents risk factors for geophagia and should be taken into account in analyses such as ours.

The risk for acute symptomatic seizures was increased by previous hospitalization, which, as explained above, represents febrile infections that are important causes of acute symptomatic seizures.^17^ Family history of seizures is widely recognized as a risk factor for febrile seizures and suggests possible genetic susceptibility to acute symptomatic seizures.^35^ Acute symptomatic seizures preceded the onset of epilepsy in 8% of children, consistent with previous observations that the risk for epilepsy is increased in those with acute symptomatic seizures compared with those without seizures.^25^

Focal seizures semiology was common, in similar proportions with previous studies in Africa, and likely represent identifiable and preventable underlying causes of epilepsy. Focal with impaired awareness seizures, previously known as complex partial seizures, were common. This is perhaps an indication that temporal lobe epilepsy is common in this area. We have observed unilateral and bilateral mesial temporal sclerosis in more than one‐third of patients with epilepsy using magnetic resonance imaging, which we think is related to febrile status epilepticus.^36^ The underlying symptomatic causes can be identified through neuroimaging, which was unavailable in our setting, and might have provided insights into treatment and prognosis of epilepsy. The commonest generalized seizure types, such as tonic, tonic–clonic, atonic, and myoclonic, often presented together, as in epilepsy syndromes of older children, for example, Lennox–Gastaut syndrome.

Overall, 83% of children were not receiving AEDs, similar to previous estimates (89%).^5^ Although the treatment gap across all age groups has been substantially reduced in this area^31^, ^37^ following educational interventions, it is still significantly greater in children than adults.^2^ Use of AEDs and adherence in children is decided by parents, who choose treatment options for children,^38^ and epilepsy in children may go untreated if mistaken for febrile seizures. The nonadherence to treatment in childhood epilepsy explains the high rates of status epilepticus, similar in previous studies in this area.^39^ Daily seizure frequency was commonly reported, particularly in those with active epilepsy, as would be expected. Adherence to treatment may control seizure frequency and perhaps improve outcome.

EEG was abnormal in 39%, higher than that reported in a previous study (20%),^5^ but comparable to African estimates.^12^ Focal abnormalities were particularly common, suggesting identifiable underlying symptomatic causes. The proportion of generalized seizure semiology with focal features was 13%, less than the previous rates of 29% in all age groups,^39^ suggesting improved concordance between the EEG and reported seizure types in the present study. The characteristic posterior parietal–occipital EEG abnormalities may be related to damage to regions supplied by middle and posterior cerebral arteries, as they were more frequent in perinatal complication or nontraumatic acute encephalopathies,^40^ which rules out benign epilepsy with occipital paroxysms. We hoped to use EEG to identify epilepsy syndromes, but this was only helpful in identifying childhood absence and Lennox–Gastaut epilepsies, implying that future detailed EEG studies, for example, sleep‐induced and passive eye‐closure EEG, may be helpful. Focal seizures were more common in those who had an EEG recorded; this could be a reflection of the preponderance of focal seizures in the entire epilepsy sample rather than a bias.

The overall prevalence of neurobehavioral problems of 54% in children with epilepsy is slightly higher than for previous similar studies of behavioral problems in the area (49%)^7^; this study did not include ASD and ADHD, which may have overlapped with behavioral problems. Compared to community controls, epilepsy was several times more strongly associated with ADHD (OR = 15), ASD (OR = 37), cognitive impairments (OR = 15), and motor deficits (OR = 35). The associations were greatest in active epilepsy, as in a previous study,^7^ suggesting that frequent seizures may be responsible. Neurobehavioral problems taken together with generalized epileptiform EEG patterns identified 8% with possible Lennox–Gastauat syndrome, a classification that may help to optimize treatment for these children. The associations with neurobehavioral comorbidities are likely to be more complex, perhaps related to the networks of the maturing brain, household environment, or even interaction within the family or parenting processes.^41^ HIV status may have confounded the neurobehavioral outcomes, but virology tests were not routinely performed in the study. Health services for assessing and addressing these comorbidities should be set up in low and middle income countries to ensure comprehensive management of the needs of children with epilepsy.

It is thought that neurobehavioral comorbidities of epilepsy may have more detrimental impact on the quality of life of the child than seizures.^7^ Therefore, neurobehavioral comorbidities should be assessed in children with epilepsy, through training of clinicians and development of locally appropriate assessment tools. Comprehensive management programs for children with epilepsy that address neurobehavioral comorbidity should be developed in resource‐limited settings.

This is a population‐based study in which detailed clinical and neuropsychological evaluations were performed in stage II after positive screening in stage I. Systematic assessment of both epilepsy and acute symptomatic seizures ensured that these two conditions were well differentiated, avoiding overestimation of either. All tools are standardized and underwent adaptation and validation before their application in the study. A detailed investigation of risk factors was informed by a thorough search of the literature. There was attrition between stages I and II, although we accounted for this in the adjusted prevalence. Lack of prolonged EEG recording, sleep EEG studies, and telemetry may have limited identification of epilepsy syndromes. This study did not perform additional neuroimaging, metabolic, or genetic investigations. Residual confounding of unmeasured factors cannot be ruled out, and the causal role of the investigated risk factors was difficult to establish in a cross‐sectional study. These results are only generalizable in other settings with similar risk factors.

This study shows that the burden of seizure disorders in this rural area of Kenya is higher than in many high‐income country settings. The reduction in malaria witnessed in previous studies may have reduced the burden of epilepsy. Perinatal complications and previous hospital admissions with febrile infections remain important risk factors for seizure disorders in this area. The emergence of eating soil and snoring as risk factors in this and other recent studies warrants further investigation. Most epilepsy was associated with focal features, pointing to identifiable symptomatic causes. More than 80% were not receiving AEDs, urging renewed efforts in reducing the treatment gap in children to improve outcome. Epilepsy was associated with neurobehavioral comorbidities that should be addressed through comprehensive clinical services.

## Disclosure

The authors declare no conflicts of interest. We confirm that we have read the Journal's position on issues involved in ethical publication and affirm that this report is consistent with those guidelines.

## Supporting information


**TableS1.**Electroencephalographic features in those diagnosed with epilepsy.
**Table S2.** Examining whether acute seizures or epilepsy are a risk factor for snoring.
**Figure S1.** Raw prevalence of epilepsy and acute seizures in 2015.
**Figure S2.** Electroencephalogram of a 7‐year‐old female shows a clear 3‐Hz spike and slow wave pattern typical of childhood absence epilepsy.
**Appendix S1.** Members of the NDD study group.
