# Supplementary material for: Prevalence, risk factors, and neurobehavioral comorbidities of epilepsy in Kenyan children
Source: Epilepsia Open. 2017 Aug 19;2(4):388–99. doi: 10.1002/epi4.12069 (PMC5862110; doi:10.1002/epi4.12069)
Supplement: Supplementary file 1 — TableS1.Electroencephalographic features in those diagnosed with epilepsy. Table S2. Examining whether acute seizures or epilepsy are a risk factor for snoring. Figure S1. Raw prevalence of epilepsy and acute seizures in 2015. Figure S2. Electroencephalogram of a 7‐year‐old female shows a clear 3‐Hz spike and slow wave pattern typical of childhood absence epilepsy. Appendix S1. Members of the NDD study group. [file EPI4-2-388-s001.docx]

| **Supplementary table 1**. EEG features in those diagnosed with epilepsy. | | | | |
| --- | --- | --- | --- | --- |
|  | Lifetime epilepsy (%) ^a^  N=64 | Active epilepsy (%)  N=42 | Inactive epilepsy (%)  N=22 | X^2^ p-value ^b^ |
| Abnormal background | 3 (4.7) | 3 (7.1) | 0 (0) | 0.697 ^f^ |
| All focal features | 13 (20.3) | 8 (19.1) | 5 (22.7) | 0.728 |
| Temporal focal features | 3 (4.7) | 3 (7.1) | 0 (0) | 0.545 ^f^ |
| Parietal-occipital focal features | 4 (6.3) | 3 (7.1) | 1 (4.5) | 1.000 ^f^ |
| All epileptiform discharges | 22 (34.4) | 16 (38.1) | 6 (27.3) | 0.387 |
| Focal | 8 (12.5) | 6 (14.3) | 2 (9.1) | 0.704 ^f^ |
| Multifocal | 10 (15.6) | 6 (14.3) | 4 (18.2) | 0.726 ^f^ |
| Generalised | 14 (21.9) | 10 (23.8) | 4 (18.2) | 0.755 ^f^ |
| Overall abnormality | 25 (39.1) | 17 (40.5) | 8 (36.4) | 0.749 |
| ^a^ Includes both active and inactive epilepsy.  ^b^Comparisons are between active and inactive epilepsy.  ^f^ Fisher’s exact test used when any value for this variable < 5.  EEG, electroencephalography | | | | |

| **Supplementary table 2**. Examining if acute seizures or epilepsy are a risk factor for snoring | | | | |
| --- | --- | --- | --- | --- |
| Risk factor | Odds ratio | Lower 95% CI | Upper 95% CI | p-value |
| Child age | 1.28 | 0.78 | 2.09 | 0.3319 |
| Male sex | 0.93 | 0.38 | 2.29 | 0.8764 |
| Acute seizures | 0.73 | 0.25 | 2.14 | 0.5710 |
| Lifetime epilepsy | 1 | 1 | 1 |  |
| Eats cassava | 2.06 | 0.16 | 26.32 | 0.5789 |
| Eats soil | 3.33 | 0.67 | 16.43 | 0.14038 |
| Dad dead | 5.92 | 0.66 | 53.09 | 0.1119 |
| Uses bed nets | 13.43 | 0.62 | 291.11 | 0.0978 |
| Home delivery | 1.38 | 0.51 | 3.69 | 0.5274 |
| CI, confidence interval  Snoring was considered as a dependent or response variable in this analysis. | | | | |

**Supplementary figure 1. Raw prevalence of epilepsy and acute seizures in 2015.^a^ Includes lifetime and active epilepsy.**

Screened n=11223

Positive stage I, n=2361

Seen by clinician, n=1640

Total for epilepsy, n= 98

Negative stage I, n=8862

Seen by clinician, n=502

Epilepsy ^a^, n=5

Acute seizures, n=18

Epilepsy ^a^, n=93

Acute seizures, n=290

Total for acute seizures, n= 308

**Supplementary figure 2. EEG of a 7-year-old female shows a clear 3Hz spike and slow wave pattern typical of Childhood Absence Epilepsy. Montage longitudinal, LF 1.0Hz, Notch, HF 35.0Hz. sensitivity (750μVp-p).**

**
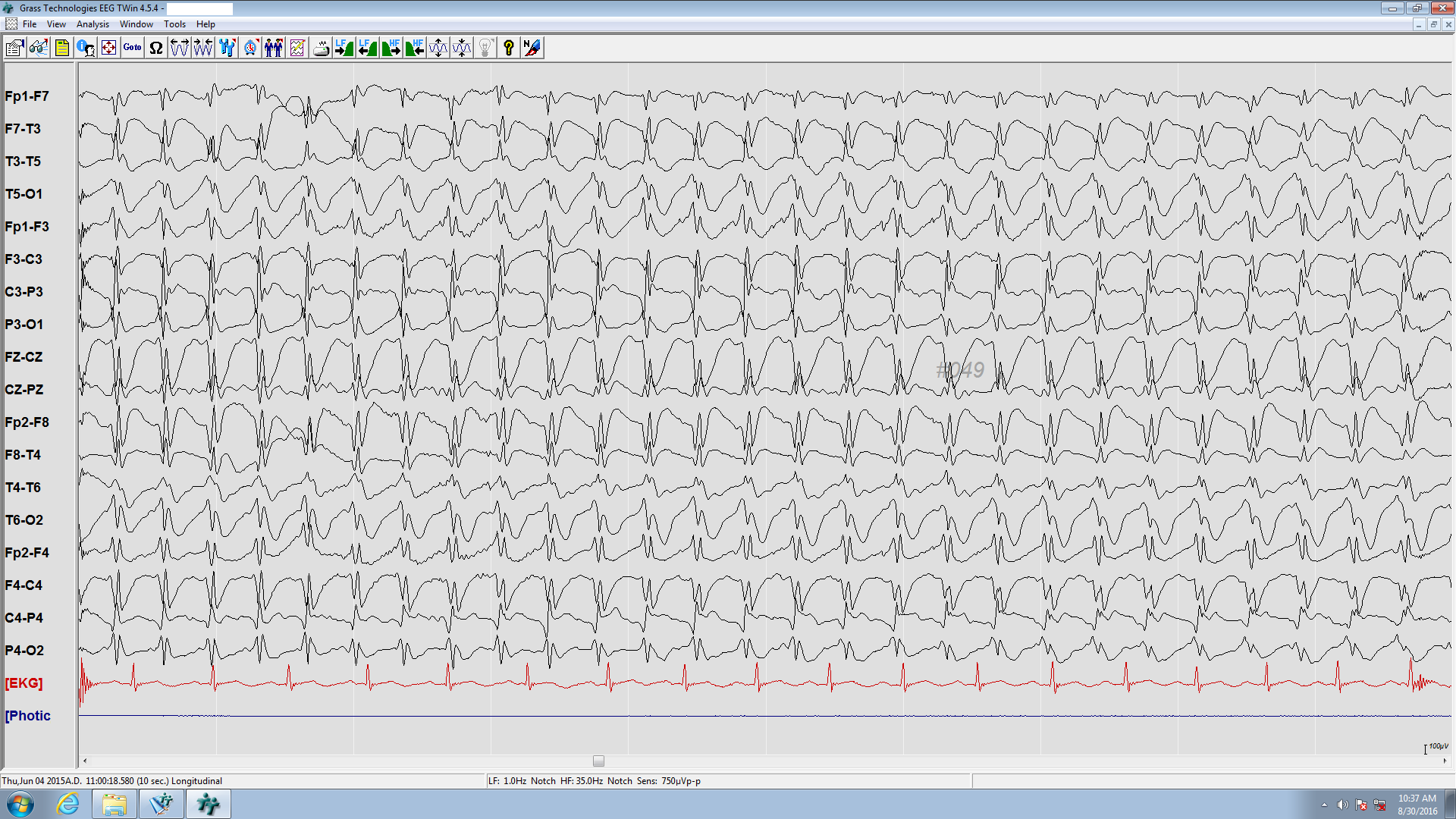
**

**Supplemtary apendix 1. Members of NDD study group**

**From KEMRI-Wellcome Trust Research Programme**

Prof Charles Newton; Dr Symon Kariuki; Dr Amina Abubakar; Fredrick Ibinda; Joseph Gona; Rachael Odhiambo; Martha Kombe; and Michael Kazungu.

**From Institute of Psychiatry, Psychology and Neuroscience at Kings College London**

Dr Jacqueline Philips-Owen
